# Methyl (2*E*)-2-({2-[(2*E*)-2-benzyl­idene-3-meth­oxy-3-oxoprop­yl]-1,3-dioxoindan-2-yl}meth­yl)-3-phenyl­prop-2-enoate

**DOI:** 10.1107/S1600536812017965

**Published:** 2012-04-28

**Authors:** D. Lakshmanan, S. Murugavel, D. Kannan, M. Bakthadoss

**Affiliations:** a Department of Physics, C. Abdul Hakeem College of Engineering & Technology, Melvisharam, Vellore 632 509, India; b Department of Physics, Thanthai Periyar Government Institute of Technology, Vellore 632 002, India; c Department of Organic Chemistry, University of Madras, Maraimalai Campus, Chennai 600 025, India

## Abstract

In the title compound, C_31_H_26_O_6_, the five-membered ring of the indane unit adopts a slight envelope conformation with the flap atom displaced by 1.38 (14) Å. The mol­ecular conformation is stabilized by an intra­molecular C—H⋯O hydrogen bond, which generates an *S*(9) ring motif. In the crystal, pairs of C—H⋯O hydrogen bonds link centrosymmetrically related mol­ecules into dimers, generating *R*_2_^2^(22) ring motifs. The crystal packing is further stabilized by C—H⋯π inter­actions.

## Related literature

Indene ring systems are present in a large number of biologi­cally active compounds, and their metallocene complexes are able to catalyse olefin polymerization, see: Rayabarapu *et al.* (2003 ▶); Senanayake *et al.* (1995 ▶). For ring puckering analysis, see: Cremer & Pople (1975 ▶). For hydrogen-bond motifs, see: Bernstein *et al.* (1995 ▶).
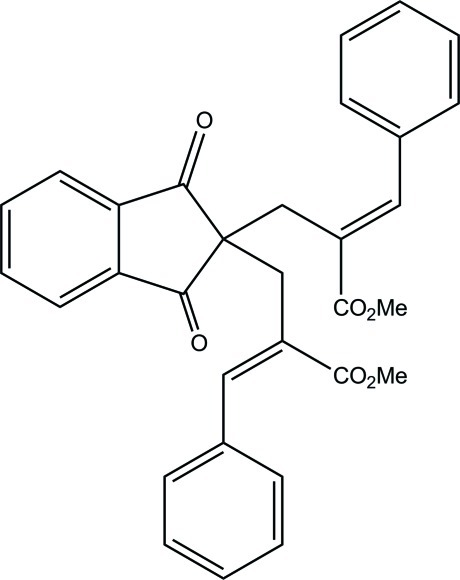


## Experimental

### 

#### Crystal data

C_31_H_26_O_6_
*M* *_r_* = 494.52
Triclinic, 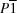

*a* = 10.5657 (4) Å
*b* = 10.9275 (5) Å
*c* = 11.8961 (5) Å
α = 71.250 (2)°
β = 77.889 (3)°
γ = 76.656 (2)°
*V* = 1251.70 (9) Å^3^
*Z* = 2
Mo *K*α radiation
μ = 0.09 mm^−1^
*T* = 293 K
0.25 × 0.23 × 0.17 mm

#### Data collection

Bruker APEXII CCD diffractometer
Absorption correction: multi-scan (*SADABS*; Sheldrick, 1996 ▶) *T*_min_ = 0.978, *T*_max_ = 0.985
25995 measured reflections
6921 independent reflections
5016 reflections with *I* > 2σ(*I*)
*R*_int_ = 0.028

#### Refinement

*R*[*F* ^2^ > 2σ(*F* ^2^)] = 0.044
*wR*(*F* ^2^) = 0.128
*S* = 1.04
6921 reflections
337 parameters
H-atom parameters constrained
Δρ_max_ = 0.28 e Å^−3^
Δρ_min_ = −0.18 e Å^−3^

### 

Data collection: *APEX2* (Bruker, 2004 ▶); cell refinement: *APEX2* and *SAINT* (Bruker, 2004 ▶); data reduction: *SAINT* and *XPREP* (Bruker, 2004 ▶); program(s) used to solve structure: *SHELXS97* (Sheldrick, 2008 ▶); program(s) used to refine structure: *SHELXL97* (Sheldrick, 2008 ▶); molecular graphics: *ORTEP-3* (Farrugia (1997 ▶); software used to prepare material for publication: *SHELXL97* and *PLATON* (Spek, 2009 ▶).

## Supplementary Material

Crystal structure: contains datablock(s) global, I. DOI: 10.1107/S1600536812017965/bt5874sup1.cif

Structure factors: contains datablock(s) I. DOI: 10.1107/S1600536812017965/bt5874Isup2.hkl

Supplementary material file. DOI: 10.1107/S1600536812017965/bt5874Isup3.cml

Additional supplementary materials:  crystallographic information; 3D view; checkCIF report

## Figures and Tables

**Table 1:** Hydrogen-bond geometry (Å, °) *Cg*1 and *Cg*2 are the centroids of the C13–C18 and C24–C29 benzene rings, respectively.

*D*—H⋯*A*	*D*—H	H⋯*A*	*D*⋯*A*	*D*—H⋯*A*
C14—H14⋯O1	0.93	2.40	3.288 (2)	161
C27—H27⋯O2^i^	0.93	2.46	3.207 (2)	137
C31—H31*C*⋯*Cg*1^ii^	0.96	2.87	3.554 (2)	129
C20—H20*B*⋯*Cg*2^iii^	0.96	2.86	3.500 (2)	125

Symmetry codes: (i) 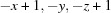; (ii) 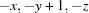; (iii) 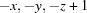.

**Fig. 1.**
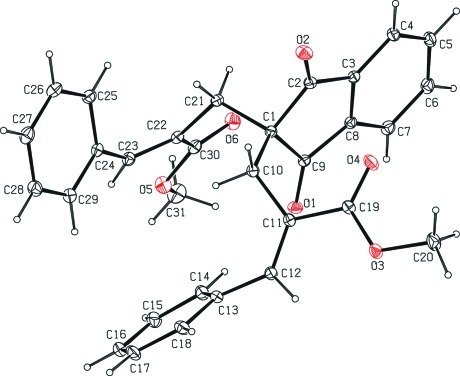
The molecular structure of the title compound with the atom numbering scheme. Displacement ellipsoids are drawn at the 10% probability level. H atoms are presented as a small cycles of arbitrary radius.

**Fig. 2.**
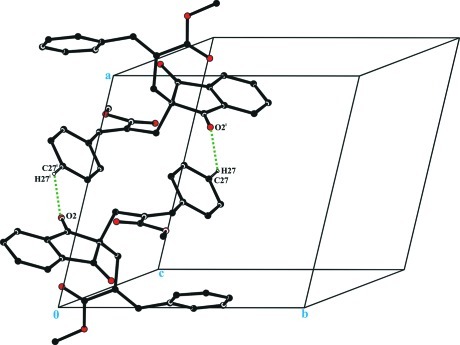
Part of the crystal structure of the title compound showing C—H···O intermolecular hydrogen bonds (dotted lines) generating R22(22) centrosymmetric dimer. [Symmetry code: (i) 1 - x, -y, 1 - z].

**Fig. 3.**
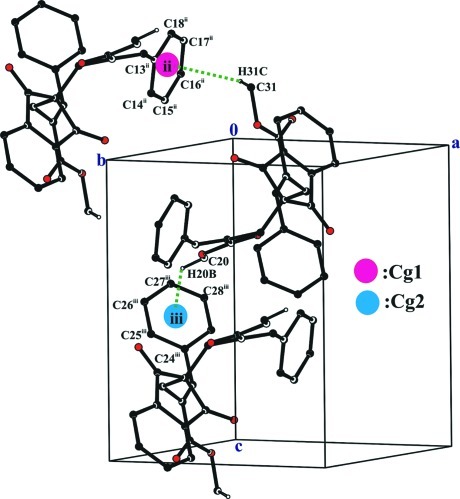
A view of the C—H···π interactions (dotted lines) in the crystal structure of the title compound. Cg1 and Cg2 denotes centroids of the C13–C18 benzene ring and C24–C29 benzene ring, respectively. [Symmetry codes: (ii) -x, 1 - y, -z; (iii) -x, -y, 1 - z].

## supplementary crystallographic information

### Comment

Indene ring frameworks are present in a large number of biologically active compounds, and their metallocene complexes are able to catalyze olefin polymerization (Senanayake *et al.*, 1995; Rayabarapu *et al.*, 2003). Some derivatives have shown analgesic and myorelaxation activity, and others are used as valuable intermediates for the synthesis of indenyl chrysanthemates that possess insecticidal properties. So in the recent three decades, many chemists have been attracted by the synthesis of indenes. In view of this biological importance, the crystal structure of the title compound has been determined and the results are presented here.

Fig. 1. shows a displacement ellipsoid plot of (I), with the atom numbering scheme. The cyclopentane (C1/C2/C3/C8/C9) ring adopts an envelope conformation with the C1 [displacement = 1.38 (14) Å] atom as the flap atom and with puckering parameters (Cremer & Pople, 1975), q_2_ = 0.1418 (13) Å and φ_2_ = 184.2 (6)°. The indene (C1–C9) moeity forms dihedral angles of 50.0 (1)° and 22.7 (1)° respectively, with the C13–C18 and C24–C29 benzene rings. The dihedral angle between two benzene rings is 65.0 (1)°.

The molecular structure is stabilized by C14—H14···O1 intramolecular hydrogen bond, forming S(9) ring motif (Bernstein *et al.*, 1995) (Table 1). In the crystal packing (Fig. 2), the molecules at *x, y, z* and *1 - x,-y,1 - z* are linked by C27—H27··· O2 hydrogen bonds into cyclic centrosymmetric *R_2_^2^*(22) dimers. The crystal packing is further stabilized by two C—H···π interactions, the first one between a methyl H31C atom and neighbouring benene ring (C13–C18), with a C31—H31C···*Cg*1^ii^ seperation of 2.87 Å (Fig. 3 and Table 1; *Cg*1 is the centroid of the C13–C18 benzene ring, Symmetry code as in Fig.3), and the second one between another methyl H20B atom and neighbouring benzene ring (C24–C29), with a C20—H20B···*Cg*2^iii^ seperation of 2.86 Å (Fig. 3 and Table 1; *Cg*2 is the centroid of the C24–C29 benzene ring, Symmetry code as in Fig.3).

### Experimental

To a stirred solution of 2,3-dihydro-1*H*-indene-1,3-dione (1 mmol, 0.146 g) and potassium carbonate (2.5 mmol, 0.345 g) was stirred for 15 minutes in acetonitrile as solvent at room temperature. To this solution, methyl (2*Z*)-2-(bromomethyl) -3-phenylprop-2-enoate (2 mmol, 0.510 g) was added till the addition is complete. After the completion of the reaction as indicated by TLC, acetonitrile solvent was evaporated. Ethylacetate (15 ml) and water (15 ml) were added to the crude mass. The organic layer was dried over anhydrous sodium sulfate. Removal of solvent led to the crude product. The pure title compound was obtained as a colorless solid (0.475 g, 96% yield). Recrystallization was carried out using ethylacetate as solvent.

### Refinement

H atoms were positioned geometrically, with C—H = 0.93–0.98 Å and constrained to ride on their parent atom, with *U*_iso_(H)=1.5*U*_eq_ for methyl H atoms and 1.2*U*_eq_(C) for other H atoms.

### Crystal data

**Table:**

C_31_H_26_O_6_	*Z* = 2
*M**_r_* = 494.52	*F*(000) = 520
Triclinic, *P*1	*D*_x_ = 1.312 Mg m^−^^3^
Hall symbol: -P 1	Mo *K*α radiation, λ = 0.71073 Å
*a* = 10.5657 (4) Å	Cell parameters from 7169 reflections
*b* = 10.9275 (5) Å	θ = 2.0–29.8°
*c* = 11.8961 (5) Å	µ = 0.09 mm^−^^1^
α = 71.250 (2)°	*T* = 293 K
β = 77.889 (3)°	Block, colourless
γ = 76.656 (2)°	0.25 × 0.23 × 0.17 mm
*V* = 1251.70 (9) Å^3^	

### Data collection

**Table:**

Bruker APEXII CCD diffractometer	6921 independent reflections
Radiation source: fine-focus sealed tube	5016 reflections with *I* > 2σ(*I*)
Graphite monochromator	*R*_int_ = 0.028
Detector resolution: 10.0 pixels mm^-1^	θ_max_ = 29.8°, θ_min_ = 2.0°
ω scans	*h* = −14→14
Absorption correction: multi-scan (*SADABS*; Sheldrick, 1996)	*k* = −15→15
*T*_min_ = 0.978, *T*_max_ = 0.985	*l* = −12→16
25995 measured reflections	

### Refinement

**Table:**

Refinement on *F*^2^	Secondary atom site location: difference Fourier map
Least-squares matrix: full	Hydrogen site location: inferred from neighbouring sites
*R*[*F*^2^ > 2σ(*F*^2^)] = 0.044	H-atom parameters constrained
*wR*(*F*^2^) = 0.128	*w* = 1/[σ^2^(*F*_o_^2^) + (0.0592*P*)^2^ + 0.201*P*] where *P* = (*F*_o_^2^ + 2*F*_c_^2^)/3
*S* = 1.04	(Δ/σ)_max_ < 0.001
6921 reflections	Δρ_max_ = 0.28 e Å^−^^3^
337 parameters	Δρ_min_ = −0.18 e Å^−^^3^
0 restraints	Extinction correction: *SHELXL97* (Sheldrick, 2008), Fc^*^=kFc[1+0.001xFc^2^λ^3^/sin(2θ)]^-1/4^
Primary atom site location: structure-invariant direct methods	Extinction coefficient: 0.0071 (18)

### Special details

**Table:**

Geometry. All e.s.d.'s (except the e.s.d. in the dihedral angle between two l.s. planes) are estimated using the full covariance matrix. The cell e.s.d.'s are taken into account individually in the estimation of e.s.d.'s in distances, angles and torsion angles; correlations between e.s.d.'s in cell parameters are only used when they are defined by crystal symmetry. An approximate (isotropic) treatment of cell e.s.d.'s is used for estimating e.s.d.'s involving l.s. planes.
Refinement. Refinement of *F*^2^ against ALL reflections. The weighted *R*-factor *wR* and goodness of fit *S* are based on *F*^2^, conventional *R*-factors *R* are based on *F*, with *F* set to zero for negative *F*^2^. The threshold expression of *F*^2^ > σ(*F*^2^) is used only for calculating *R*-factors(gt) *etc*. and is not relevant to the choice of reflections for refinement. *R*-factors based on *F*^2^ are statistically about twice as large as those based on *F*, and *R*- factors based on ALL data will be even larger.

### Fractional atomic coordinates and isotropic or equivalent isotropic displacement parameters (Å^2^)

**Table:**

	*x*	*y*	*z*	*U*_iso_*/*U*_eq_	
C1	0.25024 (11)	0.03216 (11)	0.19693 (10)	0.0334 (2)	
C2	0.29043 (12)	−0.11776 (12)	0.22493 (11)	0.0379 (3)	
C3	0.27268 (11)	−0.15476 (12)	0.12061 (11)	0.0357 (3)	
C4	0.31247 (13)	−0.27427 (13)	0.09515 (13)	0.0441 (3)	
H4	0.3578	−0.3463	0.1468	0.053*	
C5	0.28266 (14)	−0.28279 (15)	−0.00914 (14)	0.0508 (4)	
H5	0.3097	−0.3616	−0.0288	0.061*	
C6	0.21335 (15)	−0.17655 (16)	−0.08524 (14)	0.0525 (4)	
H6	0.1929	−0.1857	−0.1541	0.063*	
C7	0.17414 (13)	−0.05732 (14)	−0.06069 (12)	0.0455 (3)	
H7	0.1277	0.0142	−0.1120	0.055*	
C8	0.20613 (11)	−0.04741 (12)	0.04293 (11)	0.0352 (3)	
C9	0.17798 (11)	0.06785 (11)	0.08894 (10)	0.0335 (2)	
C10	0.17153 (12)	0.08121 (12)	0.30306 (11)	0.0364 (3)	
H10A	0.2062	0.0235	0.3749	0.044*	
H10B	0.1909	0.1673	0.2916	0.044*	
C11	0.02411 (12)	0.09234 (11)	0.32861 (10)	0.0344 (2)	
C12	−0.05926 (13)	0.20136 (12)	0.33956 (12)	0.0395 (3)	
H12	−0.1460	0.1918	0.3706	0.047*	
C13	−0.03082 (12)	0.33552 (12)	0.30845 (12)	0.0404 (3)	
C14	0.02253 (14)	0.39710 (14)	0.19283 (14)	0.0496 (3)	
H14	0.0492	0.3504	0.1364	0.060*	
C15	0.03664 (16)	0.52755 (15)	0.16017 (17)	0.0594 (4)	
H15	0.0699	0.5686	0.0815	0.071*	
C16	0.00162 (16)	0.59609 (15)	0.24355 (18)	0.0610 (4)	
H16	0.0123	0.6832	0.2221	0.073*	
C17	−0.04925 (17)	0.53541 (15)	0.35889 (17)	0.0610 (4)	
H17	−0.0714	0.5814	0.4158	0.073*	
C18	−0.06809 (16)	0.40642 (14)	0.39177 (14)	0.0517 (3)	
H18	−0.1056	0.3675	0.4695	0.062*	
C19	−0.02906 (13)	−0.02882 (12)	0.34965 (11)	0.0389 (3)	
C20	−0.21535 (18)	−0.13120 (16)	0.41099 (18)	0.0694 (5)	
H20A	−0.2102	−0.1496	0.3363	0.104*	
H20B	−0.3058	−0.1147	0.4460	0.104*	
H20C	−0.1688	−0.2052	0.4648	0.104*	
C21	0.38551 (12)	0.08067 (12)	0.16164 (12)	0.0387 (3)	
H21A	0.4251	0.0591	0.2337	0.046*	
H21B	0.4434	0.0330	0.1089	0.046*	
C22	0.37728 (11)	0.22488 (13)	0.10063 (11)	0.0379 (3)	
C23	0.38335 (13)	0.31646 (13)	0.15049 (12)	0.0411 (3)	
H23	0.3673	0.4022	0.1013	0.049*	
C24	0.41114 (12)	0.30482 (12)	0.27018 (12)	0.0392 (3)	
C25	0.51220 (13)	0.21207 (14)	0.32403 (13)	0.0459 (3)	
H25	0.5591	0.1478	0.2881	0.055*	
C26	0.54314 (14)	0.21504 (16)	0.43022 (14)	0.0529 (4)	
H26	0.6109	0.1528	0.4650	0.063*	
C27	0.47525 (16)	0.30853 (17)	0.48491 (14)	0.0569 (4)	
H27	0.4971	0.3102	0.5561	0.068*	
C28	0.37445 (16)	0.39995 (16)	0.43375 (14)	0.0549 (4)	
H28	0.3275	0.4632	0.4709	0.066*	
C29	0.34272 (14)	0.39827 (13)	0.32762 (13)	0.0463 (3)	
H29	0.2744	0.4607	0.2939	0.056*	
C30	0.36793 (13)	0.26335 (14)	−0.02990 (12)	0.0437 (3)	
C31	0.3415 (2)	0.4347 (2)	−0.20839 (15)	0.0730 (5)	
H31A	0.4247	0.4020	−0.2489	0.110*	
H31B	0.3243	0.5289	−0.2364	0.110*	
H31C	0.2732	0.4010	−0.2247	0.110*	
O1	0.10725 (9)	0.17082 (9)	0.05059 (8)	0.0454 (2)	
O2	0.33508 (11)	−0.18975 (10)	0.31315 (9)	0.0565 (3)	
O3	−0.15730 (10)	−0.01759 (9)	0.38974 (10)	0.0549 (3)	
O4	0.03542 (11)	−0.12708 (10)	0.32961 (12)	0.0659 (3)	
O5	0.34473 (12)	0.39300 (10)	−0.08061 (9)	0.0581 (3)	
O6	0.38424 (11)	0.18503 (11)	−0.08508 (9)	0.0588 (3)	

### Atomic displacement parameters (Å^2^)

**Table:**

	*U*^11^	*U*^22^	*U*^33^	*U*^12^	*U*^13^	*U*^23^
C1	0.0328 (5)	0.0355 (6)	0.0339 (6)	−0.0033 (4)	−0.0080 (5)	−0.0124 (5)
C2	0.0363 (6)	0.0370 (6)	0.0400 (7)	−0.0031 (5)	−0.0089 (5)	−0.0107 (5)
C3	0.0314 (5)	0.0373 (6)	0.0389 (6)	−0.0072 (5)	−0.0020 (5)	−0.0129 (5)
C4	0.0413 (7)	0.0373 (6)	0.0543 (8)	−0.0094 (5)	−0.0011 (6)	−0.0160 (6)
C5	0.0496 (8)	0.0499 (8)	0.0620 (9)	−0.0160 (6)	0.0051 (7)	−0.0316 (7)
C6	0.0507 (8)	0.0688 (10)	0.0513 (8)	−0.0167 (7)	−0.0036 (6)	−0.0337 (8)
C7	0.0428 (7)	0.0575 (8)	0.0408 (7)	−0.0080 (6)	−0.0090 (6)	−0.0192 (6)
C8	0.0314 (6)	0.0405 (6)	0.0357 (6)	−0.0072 (5)	−0.0033 (5)	−0.0141 (5)
C9	0.0301 (5)	0.0375 (6)	0.0323 (6)	−0.0052 (4)	−0.0038 (4)	−0.0101 (5)
C10	0.0394 (6)	0.0395 (6)	0.0335 (6)	−0.0062 (5)	−0.0070 (5)	−0.0142 (5)
C11	0.0402 (6)	0.0346 (6)	0.0295 (6)	−0.0086 (5)	−0.0023 (5)	−0.0109 (5)
C12	0.0383 (6)	0.0384 (6)	0.0428 (7)	−0.0099 (5)	0.0010 (5)	−0.0153 (5)
C13	0.0372 (6)	0.0356 (6)	0.0507 (7)	−0.0030 (5)	−0.0072 (5)	−0.0174 (6)
C14	0.0503 (8)	0.0433 (7)	0.0570 (9)	−0.0119 (6)	0.0022 (6)	−0.0205 (6)
C15	0.0567 (9)	0.0449 (8)	0.0729 (11)	−0.0169 (7)	0.0007 (8)	−0.0130 (7)
C16	0.0551 (9)	0.0371 (7)	0.0962 (13)	−0.0076 (6)	−0.0189 (9)	−0.0217 (8)
C17	0.0724 (10)	0.0440 (8)	0.0787 (12)	0.0041 (7)	−0.0265 (9)	−0.0344 (8)
C18	0.0603 (9)	0.0432 (7)	0.0536 (8)	0.0021 (6)	−0.0142 (7)	−0.0207 (6)
C19	0.0453 (7)	0.0360 (6)	0.0339 (6)	−0.0095 (5)	0.0016 (5)	−0.0111 (5)
C20	0.0665 (10)	0.0526 (9)	0.0910 (13)	−0.0333 (8)	0.0190 (9)	−0.0265 (9)
C21	0.0317 (6)	0.0418 (6)	0.0442 (7)	−0.0032 (5)	−0.0073 (5)	−0.0155 (5)
C22	0.0298 (5)	0.0454 (7)	0.0386 (6)	−0.0076 (5)	−0.0044 (5)	−0.0118 (5)
C23	0.0407 (6)	0.0418 (7)	0.0405 (7)	−0.0106 (5)	−0.0073 (5)	−0.0085 (5)
C24	0.0365 (6)	0.0425 (7)	0.0405 (7)	−0.0140 (5)	−0.0049 (5)	−0.0100 (5)
C25	0.0363 (6)	0.0542 (8)	0.0496 (8)	−0.0072 (6)	−0.0054 (6)	−0.0192 (6)
C26	0.0405 (7)	0.0676 (9)	0.0510 (8)	−0.0086 (6)	−0.0135 (6)	−0.0139 (7)
C27	0.0559 (9)	0.0772 (11)	0.0461 (8)	−0.0196 (8)	−0.0102 (7)	−0.0223 (8)
C28	0.0583 (9)	0.0600 (9)	0.0536 (9)	−0.0114 (7)	−0.0029 (7)	−0.0286 (7)
C29	0.0455 (7)	0.0440 (7)	0.0509 (8)	−0.0093 (6)	−0.0072 (6)	−0.0144 (6)
C30	0.0355 (6)	0.0536 (8)	0.0428 (7)	−0.0125 (5)	−0.0025 (5)	−0.0135 (6)
C31	0.0816 (12)	0.0871 (13)	0.0457 (9)	−0.0209 (10)	−0.0202 (8)	−0.0016 (9)
O1	0.0458 (5)	0.0430 (5)	0.0455 (5)	0.0049 (4)	−0.0157 (4)	−0.0133 (4)
O2	0.0703 (7)	0.0465 (6)	0.0492 (6)	0.0059 (5)	−0.0277 (5)	−0.0085 (5)
O3	0.0466 (5)	0.0408 (5)	0.0757 (7)	−0.0176 (4)	0.0115 (5)	−0.0207 (5)
O4	0.0585 (6)	0.0452 (6)	0.0961 (9)	−0.0152 (5)	0.0167 (6)	−0.0367 (6)
O5	0.0748 (7)	0.0553 (6)	0.0427 (6)	−0.0113 (5)	−0.0180 (5)	−0.0061 (5)
O6	0.0670 (7)	0.0698 (7)	0.0467 (6)	−0.0169 (5)	−0.0023 (5)	−0.0268 (5)

### Geometric parameters (Å, º)

**Table:**

C1—C9	1.5289 (16)	C17—C18	1.386 (2)
C1—C10	1.5368 (17)	C17—H17	0.9300
C1—C2	1.5370 (17)	C18—H18	0.9300
C1—C21	1.5668 (16)	C19—O4	1.1970 (15)
C2—O2	1.2035 (15)	C19—O3	1.3297 (16)
C2—C3	1.4812 (17)	C20—O3	1.4374 (17)
C3—C8	1.3855 (17)	C20—H20A	0.9600
C3—C4	1.3862 (17)	C20—H20B	0.9600
C4—C5	1.378 (2)	C20—H20C	0.9600
C4—H4	0.9300	C21—C22	1.4985 (18)
C5—C6	1.383 (2)	C21—H21A	0.9700
C5—H5	0.9300	C21—H21B	0.9700
C6—C7	1.378 (2)	C22—C23	1.3359 (18)
C6—H6	0.9300	C22—C30	1.4911 (19)
C7—C8	1.3868 (17)	C23—C24	1.4735 (18)
C7—H7	0.9300	C23—H23	0.9300
C8—C9	1.4752 (17)	C24—C29	1.3902 (19)
C9—O1	1.2073 (14)	C24—C25	1.3957 (19)
C10—C11	1.5070 (17)	C25—C26	1.381 (2)
C10—H10A	0.9700	C25—H25	0.9300
C10—H10B	0.9700	C26—C27	1.369 (2)
C11—C12	1.3339 (17)	C26—H26	0.9300
C11—C19	1.4841 (17)	C27—C28	1.376 (2)
C12—C13	1.4779 (17)	C27—H27	0.9300
C12—H12	0.9300	C28—C29	1.379 (2)
C13—C14	1.385 (2)	C28—H28	0.9300
C13—C18	1.3882 (19)	C29—H29	0.9300
C14—C15	1.387 (2)	C30—O6	1.1994 (17)
C14—H14	0.9300	C30—O5	1.3354 (17)
C15—C16	1.370 (2)	C31—O5	1.4453 (19)
C15—H15	0.9300	C31—H31A	0.9600
C16—C17	1.372 (3)	C31—H31B	0.9600
C16—H16	0.9300	C31—H31C	0.9600
			
C9—C1—C10	114.88 (10)	C16—C17—H17	119.5
C9—C1—C2	102.63 (9)	C18—C17—H17	119.5
C10—C1—C2	114.96 (10)	C17—C18—C13	119.89 (15)
C9—C1—C21	112.06 (10)	C17—C18—H18	120.1
C10—C1—C21	108.52 (9)	C13—C18—H18	120.1
C2—C1—C21	103.19 (9)	O4—C19—O3	122.35 (12)
O2—C2—C3	126.87 (12)	O4—C19—C11	123.84 (12)
O2—C2—C1	125.44 (12)	O3—C19—C11	113.78 (10)
C3—C2—C1	107.57 (10)	O3—C20—H20A	109.5
C8—C3—C4	120.86 (12)	O3—C20—H20B	109.5
C8—C3—C2	109.67 (10)	H20A—C20—H20B	109.5
C4—C3—C2	129.47 (12)	O3—C20—H20C	109.5
C5—C4—C3	117.82 (13)	H20A—C20—H20C	109.5
C5—C4—H4	121.1	H20B—C20—H20C	109.5
C3—C4—H4	121.1	C22—C21—C1	114.63 (10)
C4—C5—C6	121.34 (13)	C22—C21—H21A	108.6
C4—C5—H5	119.3	C1—C21—H21A	108.6
C6—C5—H5	119.3	C22—C21—H21B	108.6
C7—C6—C5	121.08 (13)	C1—C21—H21B	108.6
C7—C6—H6	119.5	H21A—C21—H21B	107.6
C5—C6—H6	119.5	C23—C22—C30	119.34 (12)
C6—C7—C8	117.83 (13)	C23—C22—C21	126.49 (12)
C6—C7—H7	121.1	C30—C22—C21	114.10 (11)
C8—C7—H7	121.1	C22—C23—C24	131.01 (12)
C3—C8—C7	121.04 (12)	C22—C23—H23	114.5
C3—C8—C9	110.18 (10)	C24—C23—H23	114.5
C7—C8—C9	128.78 (12)	C29—C24—C25	117.80 (12)
O1—C9—C8	126.17 (11)	C29—C24—C23	118.80 (12)
O1—C9—C1	125.91 (11)	C25—C24—C23	123.09 (12)
C8—C9—C1	107.88 (9)	C26—C25—C24	120.52 (13)
C11—C10—C1	120.19 (10)	C26—C25—H25	119.7
C11—C10—H10A	107.3	C24—C25—H25	119.7
C1—C10—H10A	107.3	C27—C26—C25	120.79 (14)
C11—C10—H10B	107.3	C27—C26—H26	119.6
C1—C10—H10B	107.3	C25—C26—H26	119.6
H10A—C10—H10B	106.9	C26—C27—C28	119.49 (14)
C12—C11—C19	118.89 (11)	C26—C27—H27	120.3
C12—C11—C10	124.08 (11)	C28—C27—H27	120.3
C19—C11—C10	116.93 (10)	C27—C28—C29	120.30 (14)
C11—C12—C13	128.02 (12)	C27—C28—H28	119.8
C11—C12—H12	116.0	C29—C28—H28	119.8
C13—C12—H12	116.0	C28—C29—C24	121.09 (13)
C14—C13—C18	118.65 (12)	C28—C29—H29	119.5
C14—C13—C12	120.66 (12)	C24—C29—H29	119.5
C18—C13—C12	120.43 (13)	O6—C30—O5	123.11 (13)
C13—C14—C15	120.79 (14)	O6—C30—C22	123.02 (13)
C13—C14—H14	119.6	O5—C30—C22	113.82 (12)
C15—C14—H14	119.6	O5—C31—H31A	109.5
C16—C15—C14	120.12 (16)	O5—C31—H31B	109.5
C16—C15—H15	119.9	H31A—C31—H31B	109.5
C14—C15—H15	119.9	O5—C31—H31C	109.5
C15—C16—C17	119.57 (14)	H31A—C31—H31C	109.5
C15—C16—H16	120.2	H31B—C31—H31C	109.5
C17—C16—H16	120.2	C19—O3—C20	116.63 (11)
C16—C17—C18	120.93 (14)	C30—O5—C31	115.67 (13)
			
C9—C1—C2—O2	−169.81 (13)	C11—C12—C13—C18	−127.96 (15)
C10—C1—C2—O2	−44.38 (17)	C18—C13—C14—C15	−1.0 (2)
C21—C1—C2—O2	73.58 (15)	C12—C13—C14—C15	173.24 (14)
C9—C1—C2—C3	13.99 (12)	C13—C14—C15—C16	2.1 (2)
C10—C1—C2—C3	139.42 (10)	C14—C15—C16—C17	−1.0 (3)
C21—C1—C2—C3	−102.62 (10)	C15—C16—C17—C18	−1.2 (2)
O2—C2—C3—C8	173.92 (13)	C16—C17—C18—C13	2.3 (2)
C1—C2—C3—C8	−9.95 (13)	C14—C13—C18—C17	−1.2 (2)
O2—C2—C3—C4	−6.3 (2)	C12—C13—C18—C17	−175.45 (13)
C1—C2—C3—C4	169.83 (12)	C12—C11—C19—O4	171.63 (14)
C8—C3—C4—C5	−0.54 (19)	C10—C11—C19—O4	−11.90 (19)
C2—C3—C4—C5	179.70 (12)	C12—C11—C19—O3	−6.35 (17)
C3—C4—C5—C6	−1.1 (2)	C10—C11—C19—O3	170.13 (11)
C4—C5—C6—C7	1.5 (2)	C9—C1—C21—C22	55.81 (13)
C5—C6—C7—C8	−0.2 (2)	C10—C1—C21—C22	−72.09 (13)
C4—C3—C8—C7	1.85 (19)	C2—C1—C21—C22	165.52 (10)
C2—C3—C8—C7	−178.35 (11)	C1—C21—C22—C23	102.49 (14)
C4—C3—C8—C9	−178.62 (11)	C1—C21—C22—C30	−80.62 (13)
C2—C3—C8—C9	1.18 (14)	C30—C22—C23—C24	−170.34 (12)
C6—C7—C8—C3	−1.47 (19)	C21—C22—C23—C24	6.4 (2)
C6—C7—C8—C9	179.09 (12)	C22—C23—C24—C29	−142.60 (15)
C3—C8—C9—O1	−169.82 (12)	C22—C23—C24—C25	44.0 (2)
C7—C8—C9—O1	9.7 (2)	C29—C24—C25—C26	−0.69 (19)
C3—C8—C9—C1	8.11 (13)	C23—C24—C25—C26	172.75 (13)
C7—C8—C9—C1	−172.40 (12)	C24—C25—C26—C27	0.2 (2)
C10—C1—C9—O1	39.12 (17)	C25—C26—C27—C28	0.5 (2)
C2—C1—C9—O1	164.60 (12)	C26—C27—C28—C29	−0.6 (2)
C21—C1—C9—O1	−85.33 (14)	C27—C28—C29—C24	0.1 (2)
C10—C1—C9—C8	−138.82 (10)	C25—C24—C29—C28	0.6 (2)
C2—C1—C9—C8	−13.33 (12)	C23—C24—C29—C28	−173.17 (13)
C21—C1—C9—C8	96.74 (11)	C23—C22—C30—O6	167.08 (13)
C9—C1—C10—C11	32.08 (15)	C21—C22—C30—O6	−10.05 (18)
C2—C1—C10—C11	−86.71 (13)	C23—C22—C30—O5	−10.50 (17)
C21—C1—C10—C11	158.36 (10)	C21—C22—C30—O5	172.37 (11)
C1—C10—C11—C12	−126.37 (13)	O4—C19—O3—C20	1.5 (2)
C1—C10—C11—C19	57.36 (15)	C11—C19—O3—C20	179.47 (13)
C19—C11—C12—C13	−171.64 (12)	O6—C30—O5—C31	−0.5 (2)
C10—C11—C12—C13	12.2 (2)	C22—C30—O5—C31	177.03 (13)
C11—C12—C13—C14	57.91 (19)		

### Hydrogen-bond geometry (Å, º)

*Cg*1 and *Cg*2 are the centroids of the C13–C18 and C24–C29 benzene rings, respectively.

**Table:**

*D*—H···*A*	*D*—H	H···*A*	*D*···*A*	*D*—H···*A*
C14—H14···O1	0.93	2.40	3.288 (2)	161
C27—H27···O2^i^	0.93	2.46	3.207 (2)	137
C31—H31*C*···*Cg*1^ii^	0.96	2.87	3.554 (2)	129
C20—H20*B*···*Cg*2^iii^	0.96	2.86	3.500 (2)	125

Symmetry codes: (i) −*x*+1, −*y*, −*z*+1; (ii) −*x*, −*y*+1, −*z*; (iii) −*x*, −*y*, −*z*+1.
